# Unequal effects of climate change and pre-existing inequalities on the mental health of global populations

**DOI:** 10.1192/bjb.2021.26

**Published:** 2021-08

**Authors:** Shuo Zhang, Isobel Braithwaite, Vishal Bhavsar, Jayati Das-Munshi

**Affiliations:** 1 South London and the Maudsley NHS Trust, UK; 2 University College London, UK; 3 King's College London, UK

**Keywords:** Social deprivation, stigma and discrimination, low- and middle-income countries, epidemiology, aetiology

## Abstract

Climate change is already having unequal effects on the mental health of individuals and communities and will increasingly compound pre-existing mental health inequalities globally. Psychiatrists have a vital part to play in improving both awareness and scientific understanding of structural mechanisms that perpetuate these inequalities, and in responding to global calls for action to promote climate justice and resilience, which are central foundations for good mental and physical health.

Climate change is affecting the physical and mental health of human populations through direct and indirect mechanisms including population displacement, forced migration, and economic and food system instability.^1^ Global action and ‘a robust response [to climate change] … will improve human health and wellbeing’^2^ and reduce associated psychological consequences.^3^

We suggest that by addressing mental health inequalities, psychiatrists can contribute to addressing climate change and its health effects. We highlight the interconnectedness of inequality, climate and ecological destruction, and adverse mental health outcomes. We go on to propose a model for mental health practice and research that promotes climate justice and resilience in the context of increasingly complex geographical, economic and sociocultural factors.

## Climate change and mental health: unequal effects, but not by chance

The COVID-19 pandemic has shown that minoritised and indigenous communities are at heightened risk from combined social and ecological shocks, including adverse weather events and pandemics, owing to vulnerability resulting from pre-existing factors such as discrimination, exclusion, poverty, land dispossession and malnutrition.^4^ There are also inequalities in exposure to ecological hazards. A study on associations between air pollution and neighbourhood characteristics in England and The Netherlands found higher concentrations in the most deprived 20% of neighbourhoods in England, with higher concentrations in both countries in neighbourhoods with >20% non-White populations, after adjustment for urbanisation and other variables.^5^

On a global scale, there is increasing recognition that climate change is compounding many health inequities and undermining pre-existing support structures which protect against poor mental health.^6^ The climate crisis has been called a racist crisis, reflecting both its disproportionate effects on Black communities and people of colour globally, as well as its evident unequal effects within countries.^7,8^ Following Hurricane Katrina, for example, Black New Orleanians faced greater stress than their White counterparts, even after adjustment for demographics, parental status, evacuation timing, home damage and job status; income had no clear effect.^9^ There was a similar pattern for post-traumatic stress disorder, which was partly but not fully explained by greater baseline mental distress.^10^

In recent years, campaigns for international action on climate change have highlighted how unjust social and economic systems, including legacies of colonialism, structural racism, and other exclusionary forces such as sexism and ableism at all levels shape vulnerability to the effects of climate change on mental and physical health. We contend, as argued by Leon Sealey-Huggins and colleagues (2018), that ‘we can only properly understand the harm being wrought by weather events and climate change by directly connecting it to broader social and political processes, of which structural racism is a central part’.^7^

Future research must further clarify the mechanisms as a result of which people with severe mental illness are more vulnerable to the adverse effects of climate change. This should include a recognition of closely interwoven vulnerabilities due to social exclusion, stigma and direct consequences of their illness or medications, as well as the effects of unequal distribution of resources at local, national and international levels, all of which affect people's and communities’ capacities to cope and adapt.

## Intersections between urban health inequalities and climate change effects

Cities are the places where most people live globally – 56% of the global population as of 2018^11^ – and are the places where people are most exposed to the adverse effects of climate change. For example, the urban heat island effect means that higher temperatures are experienced in urban areas than in more rural ones nearby, and access to places for people to cool off in, such as shady green spaces or waterways, is often more limited. The health effects of rising exposure to high temperatures are not only physical but have also been associated with increased risks of mental ill health^12^ and with suicide.^13^

Cities are also a key focus for research into mental health inequalities, particularly in relation to differences in urban versus rural rates of schizophrenia. The spatial distributions of other mental health outcomes such as suicide and self-injury are less clear cut, suggesting a greater need to further understand the influence of sociocultural and environmental characteristics of particular neighbourhoods.^14^ One example of this is how qualitative methods have begun to unpack the paradoxically low rates of direct self-harm in highly deprived areas in London.^15^

People in cities often experience the greatest exposure to some of the key contributors to climate change, including air pollution from fossil fuels and degradation of green space. Further, increased exposure to poor-quality air and green spaces often mirrors and compounds pre-existing socioeconomic inequalities. Evidence on the lifelong effects of air pollution on mental health outcomes such as depression continues to emerge.^16,17^ Exposure to air pollution and traffic noise are similarly unequal for different neighbourhoods with regard to both socioeconomic status and ethnicity, with those least likely to own a car often most likely to live in traffic-clogged and polluted areas.^18^ At the same time, good access to urban nature can help to partially mitigate the harmful effects of socioeconomic inequalities.^19^

Further, there has been greater awareness of the urgent need for more urban green space^20^ and a recognition of strong disparities in access to green space in European cities.^21^ Access to green space has been linked with reduced depression risk,^22,23^ and there is increasing recognition that social factors affecting both availability^24^ and levels of use^25^ seem to mediate the mental health effects of green and blue spaces such as parks, forests, rivers and beaches.

There is increasing evidence of biologically plausible explanations of associations between urban environmental exposures and mental health. These include the effects of traffic and air population exposure on neurodevelopmental pathways in children,^26^ neuroinflammation across the life course,^17^ and changes in arousal and stress responses associated with time spent in green space.^27^ Proposed mechanisms through which the green space's benefits may be manifest include a shift in attention, promoting curiosity, social networks, group cooperation and physical activity. However, methodological and interpretation challenges remain, for example, in understanding the importance of confounding factors such as noise pollution and general neighbourhood deprivation.^17^ Limited studies have translated these observations into robust evidence for improved mental health.^28^

There is also a lack of research from low- and middle-income countries (LMICs) on the effects of climate change on mental health. This is urgently needed to inform policy action in these contexts. In LMIC contexts, forced migration due to climate change – with the destination locations often being cities – is a significant challenge, and studies have highlighted that dislocation from one's home compromises emotional well-being related to happiness, life satisfaction, optimism for the future and spiritual contentment, even despite well-intentioned relocation programmes focused on material compensation and livelihood re-establishment.^29^

## Challenges in conceptualising complexity

For mental health, ‘the risks and impacts of climate change … are already rapidly accelerating, resulting in a number of direct, indirect, and overarching effects that disproportionally affect those who are most marginalised’.^30^ Our ability to fully appreciate and act on these vulnerabilities has been constrained by complexity on multiple levels, from the geographic scale to the interrelated nature of the underlying causal mechanisms.

These constraints have limited research into the effects of climate change on mental health, to date. Quantitative approaches have tended to focus mostly on proximate causes, which can obscure important structural and political drivers of the distribution of mental health effects of climate-related heatwaves, floods, wildfires and droughts.^31^ Studies which have found associations between heat and mental health outcomes such as suicide have also cautioned about the difficulties of ascertaining causal effects using an ecological study design.^13^

Berry et al (2018) proposed that a systems approach that accounts for interrelated and interdependent factors, forming a complex whole, is important for future research thinking and leadership around climate change and mental health.^32^ We argue that this approach should also accommodate a structural and intersectional understanding of pre-existing inequalities in mental health. This may come from approaches which address broader inter-penetrative global socioeconomic processes such as globalisation,^33^ and the syndemics model of health that focuses on ‘interacting, co-present or sequential diseases and the social and environmental factors that promote and enhance the negative effects of disease interaction’.^34^

## The role of psychiatry in understanding and addressing climate change

In 2015, our Australasian colleagues^35^ proposed the ‘CARM’ approach – to collaborate, advocate, research (and educate) and mitigate – as a framework for psychiatrists to join with a growing number of medical entities to act on climate change. We have proposed our recommendations within this same framework (Box 1), and with the emphasis that interventions need to be ‘coordinated and rooted in active hope’ to tackle the problem in a holistic and effective way.^30^ Alongside growing recognition that we should practice psychiatry more sustainably,^36^ we should acknowledge it as our professional and ethical responsibility to address the environmental, social and economic determinants of mental illness.^35^

Box 1 Framework for psychiatrists to act on climate change (adapted from the ‘CARM’ approach^32^).

- Collaborate
  - Work with disadvantaged communities so that their voices and priorities are better heard, and help them influence policy decisions
  - Build on existing multidisciplinary work across specialties, and with patients and the public, to drive meaningful change on key issues relevant to climate and mental health
- Advocate
  - For patients and communities’ mental health and well-being
  - For actions that strengthen local community resilience and tackle global injustice
  - For policies and funding for appropriate and evidence-based interventions to support and protect mental health following climate-related extreme events
- Research (and educate)
  - To improve understanding of mental health in relation to the causes of inequalities, climate vulnerability and resilience
  - Continue to build the evidence base for action, including through participatory and action research methods, and using mixed methods and systems approaches that recognise the interconnectedness and complexity of these subjects
  - Advocate for increased funding for research to improve understanding of how both structural inequalities and climate change affect mental health, including when they intersect and how we can address them in tandem
- Mitigate
  - Prioritise primary prevention for mental health across the life course
  - Strengthen mental health systems and links between healthcare services and local communities
  - Improve equity of access to quality care
  - Act within local services to reduce carbon and improve mental healthcare

### Collaborate

Psychiatry should work with disadvantaged communities to help them influence policies that may be linked to climate change, including empowering indigenous communities, implementing processes for equitable access to resources and ensuring inclusivity in long-term sustainable development policies. We should build on our multidisciplinary work with colleagues across specialties, with patients and the public, and with other health organisations. For example, psychiatrists can make use of the Royal College of Psychiatrists’ membership of the UK Climate and Health Alliance to collaboratively communicate the urgency of the situation and the case for action, and to influence policy makers, community organisations and other stakeholders to deliver meaningful change.

### Advocate

Climate change is a global problem which needs global cooperation and local action. As advocates for patients’ and communities’ mental health and well-being, psychiatrists can help to drive these objectives forwards. There is already strong evidence that the psychiatric community can use in advocating for policies and interventions which can build local community resilience and tackle global injustice. These could include but are not limited to urban planning and regeneration; increased green space; cleaner air; community food growing; liveable streets; and high-quality, low-carbon housing. Importantly, they should also include national and international policies for urgent action on climate change and protection for those harmed or displaced by it, which may further exacerbate pre-existing mental health inequalities. With an already increasing frequency of adverse weather events, there is also an important role for the psychiatric community in advocating for policies and funding support for appropriate mental health interventions in the wake of climate-driven events such as floods, major storms and wildfires, and to strengthen resilience to them.

### Research (and educate)

A better understanding of the causes of inequalities, vulnerability and climate resilience as they relate to mental health is clearly needed, requiring changes to both research and education. Alongside more established quantitative epidemiological approaches, this is likely to benefit from qualitative and narrative methods, as well as systems approaches that recognise the complexity of these interlinked causal relationships and policy challenges.^32^ We should continue to build the evidence base for action, including through participatory and action research methods that emphasise the voices, needs and priorities of those who are most climate vulnerable and which seek to tackle structural injustices. We should also advocate for increased funding for research focused on understanding and addressing the effects of structural inequalities and climate change on mental health.

### Mitigate

There are a number of areas where we can intervene early in addressing unequal climate change effects, for example, through prioritising primary prevention for mental health disorders across the life course and working upstream to address drivers of mental ill health and intervene early; strengthening mental health systems, particularly in areas of high deprivation; and strengthening links between clinical services and local communities. Against a backdrop of both national health service and governmental carbon reduction initiatives, psychiatric services must also both reduce their own emissions and improve mental healthcare, to avoid contributing further to the root causes of climate-related mental distress.

## Conclusion

The time is now for concerted action to better understand and intervene in the structures and policies that create and perpetuate social and ethnic inequalities globally and harm planetary health. Working together to create the conditions for good mental health, such as enabling equitable access to resources, services and healthy environments, will also strengthen climate resilience and health equality across society. We can no longer overlook the interconnected ecological and social crises, and psychiatrists can play a critical part in defining the fairer and healthier society of tomorrow.

## About the authors

**Shuo Zhang** is a Core Psychiatry Trainee at South London and the Maudsley NHS Trust, London, UK; **Isobel Braithwaite** is an Academic Clinical Fellow in Public Health and ST4 Public Health Registrar at UCL Institute of Health Informatics, University College London, London. UK; **Vishal Bhavsar** is a Women's Mental Health and Violence Reduction and Consultant Psychiatrist at Lambeth Council and the Department of Health Services and Population Research, Institute of Psychiatry, Psychology and Neuroscience (IOPPN), King's College London and the South London and Maudsley NHS Foundation Trust, London, UK; and **Jayati Das-Munshi** is a Clinician Scientist & Honorary Consultant Psychiatrist at the Department of Psychological Medicine, IOPPN, King's College London & South London and the Maudsley NHS Trust, London. UK.

## Data Availability

Data sharing not applicable to this article as no datasets were generated or analysed.
